# Supplementary material for: Wide-field mid-infrared single-photon upconversion imaging
Source: Nat Commun. 2022 Feb 28;13:1077. doi: 10.1038/s41467-022-28716-8 (PMC8885736; doi:10.1038/s41467-022-28716-8)
Supplement: Supplementary file 1 — Supplementary Information file [file 41467_2022_28716_MOESM1_ESM.pdf]

Wide-field mid-infrared single-photon upconversion imaging
# - Supplementary Information -

Kun Huang,[1,2,3,*] Jianan Fang,[1] Ming Yan,[1,2] E Wu,[1,2] and Heping Zeng[1,2,4,5,†]

[1] *State Key Laboratory of Precision Spectroscopy, East China Normal University, Shanghai 200062, China*

[2] *Chongqing Key Laboratory of Precision Optics, Chongqing Institute of East China Normal University, Chongqing, China*

[3] *Collaborative Innovation Center of Extreme Optics, Shanxi University, Taiyuan, Shanxi 030006, China*

[4] *Jinan Institute of Quantum Technology, Jinan, Shandong 250101, China*

[5] *Shanghai Research Center for Quantum Sciences, Shanghai 201315, China*

(Dated: December 2, 2021)

Supplementary Note 1: Details about experimental setup

The detailed schematic for the experimental setup is illustrated in Supplementary Figure 1. The whole system is comprised of two main parts: synchronous pulse preparation and frequency upconversion imaging. The involved laser pulses are from an ytterbium-doped fiber laser (YDFL) at 1030 nm and an extended cavity diode laser (ECDL) at 1550 nm. The YDFL is a home-made mode-locked fiber laser in a polarization-maintaining architecture, which can deliver picosecond pulses at the repetition rate about 21.6 MHz. The average power is augmented by two-stage fiber amplifiers. The preamplifier is a single-mode Yb-doped fiber amplifier, while the main amplifier is based on the Yb-doped photonic crystal fiber. The large mode-field diameter of the gain fiber favors to minimize the spectral distortion especially for an intensive peak intensity. The average power can reach to 14 W, which is divided into two branches by a polarization beam splitter. The splitting ratio can be controlled by varying the orientation angle of the half-wave plate. One portion is spatially combined via a dichroic mirror with the amplified light from the ECDL. The two beams are then focused into a periodically poled lithium niobate (PPLN) crystal to perform the difference-frequency generation (DFG). The mid-infrared (MIR) pulse is thus passively synchronized with the pump pulse at the 1030 nm. Notably, the continuous-wave injection can significantly reduce the threshold of the parametric downconversion. The overall conversion efficiency for the MIR generation was limited by the small duty cycle of the mode-locked YDFL, which could be substantially improved by using the coincidence-pumping scheme [1]. Additionally, the high sensitivity of the subsequently implemented upconversion imager alleviates the need for a high-power MIR illumination source. In the experiment, the MIR power reaches to 100 mW at the presence of 3-W pump power. The related characterization of involved optical pulses in the spectral and temporal domains will be given in the next section.

Then, the MIR beam size is expanded by a pair of CaF_2 lens, resulting in a diameter about 30

*Electronic address: khuang@lps.ecnu.edu.cn

†Electronic address: hpzeng@phy.ecnu.edu.cn

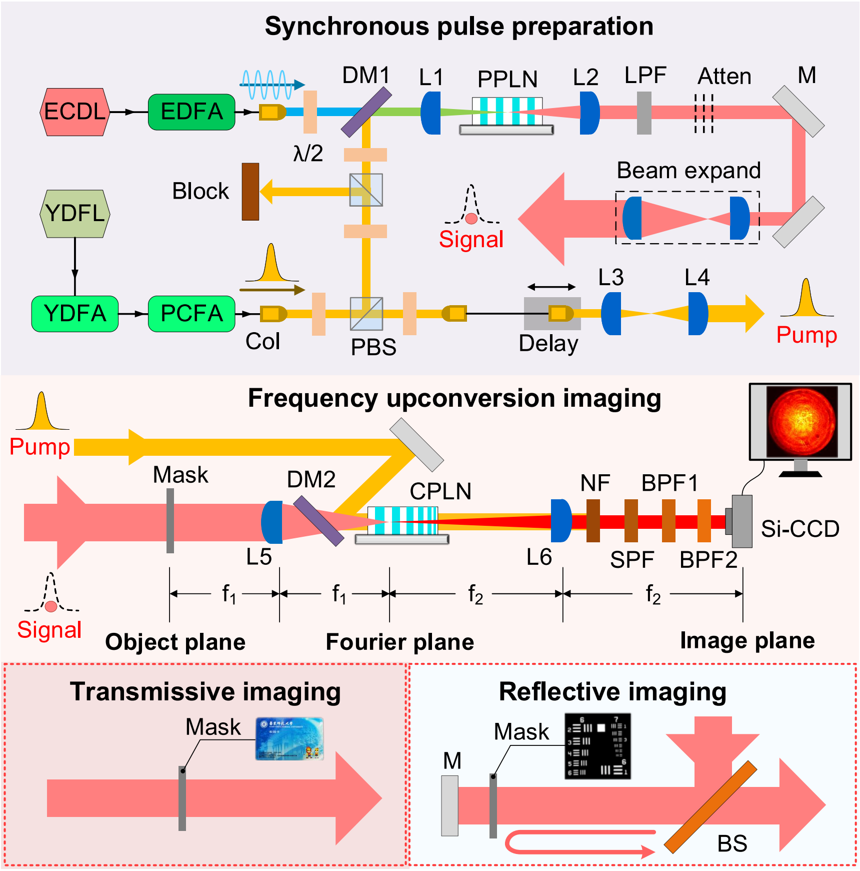

Supplementary Figure 1: Detailed schematic for the experimental setup. Two illumination modes are used to demonstrate the transmissive imaging and the reflective imaging. ECDL: extended cavity diode laser; YDFL: ytterbium-doped fiber laser; PPLN: periodically poled lithium niobate crystal; CPLN: chirped-poling niobate crystal; EDFA: Er-doped fiber amplifier; YDFA: Yb-doped fiber amplifier; PCFA: photonic crystal fiber amplifier; Col: collimator; DM: dichroic mirror; L: lens; Atten: neutral density attenuator; M: silver mirror; $\lambda/2$: half-wave plate; BS: 50/50 beam splitter; PBS: polarizing beam splitter; NF: notch filter; LPF, SPF and BPF: long-, short-, and band-pass filter.

mm. The pump beam is coupled into a fiber delay line, where one of the collimator is placed on a linear translation stage (Thorlabs, NRT150/M). The signal and pump pulses are steered into a 4f imaging system. Specifically, the illuminated mask is placed at the object plane and the silicon camera is positioned at the image plane. The MIR spatial frequency components is spectrally converted based on the sum-frequency generation (SFG) within a chirped-poling niobate (CPLN) crystal at the Fourier plane. The beam diameter of the pump waist is optimized at 1.4 mm to obtain a high spatial resolution of the upconverted image. Note that the pump size is larger than the thickness of the nonlinear crystal, which results in a cropped beam in the vertical direction. In order to suppress the background noises, the upconverted light at 771 nm passes through a series of spectral filters. The total transmission is about 80%, and the rejection ratio at the pump wavelength is estimated to be 210 dB. The high-performance filtering system is essential to achieve the single-photon sensitivity in the subsequent imaging.

To fully explore the performances of the implemented wide-field upconversion imaging system, two types of silicon-based cameras are used in the experiment. One is based on the electron multiplying CCD (EMCCD), which is featured with the single-photon sensitivity. The low dark noise of the camera allows to characterize the extremely sensitive performance of the upconversion imager [2, 3]. The other one is based on the CMOS sensor, which permits an ultra-high frame rate of 216 000 frame per second (fps). The fast camera in combination with the image upconverter enables to achieve an unprecedented frame rate in the MIR regime. Furthermore, two illumination modalities have been investigated. The transmissive imaging is employed to observe the interior structure of a campus ID card. Alternatively, the reflective illumination is used to obtain the depth information about a three-dimensional (3D) scenery. In this case, the temporal scanning of the delay line allows to the identify the reflective photons with a high-precision time stamp.

Supplementary Note 2: Characterization of upconversion imaging system

The coincidence pumping technique is used to implement the nonlinear frequency upconversion in our experiment. In this scenario, the ultrashort pump not only favors to improve the conversion efficiency due to the high peak power, but also helps to reduce the background noise within a narrow time window. Furthermore, the spectro-temporal properties of the involved optical pulses have been carefully engineered to optimize the conversion performance [4]. Supplementary Figure 2a presents the optical spectrum of the pump source, which is measured by an optical spectrum analyzer (Yokogawa, AQ6370D) with a resolution of 0.02 nm. The narrow bandwidth can preserve the spectral information of the MIR signal as much as possible, which would be desirable for the hyperspectral imaging. The ECDL (LaseGen, LTL-1500) has a narrow linewidth of $<$ 100 kHz. Consequently, the 1.8-nm pump bandwidth results in a 16-nm MIR spectrum through the DFG, as shown in Supplementary Figure 2b. In comparison to broad sources like globar or supercontinuum sources, the high-brightness MIR illumination allows to improve the overall conversion efficiency. Moreover, the resulting narrow-band SFG signal permits a tighter spectral filtering to suppress the background noise. The improved signal to noise ratio in the upconversion imaging system is essential to approach the single-photon operation.

In the temporal domain, the pulse duration of the pump τ_p is measured to be 8.5 ps as given in Supplementary Figure 2c by an auto-correlator (APE, pulseCheck). The pulse duration of the MIR signal pulse τ_s should be identical since the other beam in DFG operates at the continuous-wave mode. This assertion is verified from the width $\tau_{\rm corr}$ of the measured cross-correlation trace as shown in Supplementary Figure 2d, satisfying the relationship $\tau_{\rm corr} = \sqrt{\tau_p^2 + \tau_s^2}$. The comparable pulse width for the signal and pump fields favors to optimize the conversion efficiency. Note the $\tau_{\rm corr}$ determines the depth resolution about 1.8 mm in the 3D upconversion imaging. Additionally, the ECDL can be spectrally tuned within the telecom C-band, which allows to access MIR wavelengths from 3015 to 3170 nm as shown in Supplementary Figure 2e. The MIR spectra are recorded with the resolution of 2 nm by a grating-based spectrometer (APE, waveScan).

The peak conversion efficiency is estimated to 0.1 % at the pump power of 0.6 W, which was

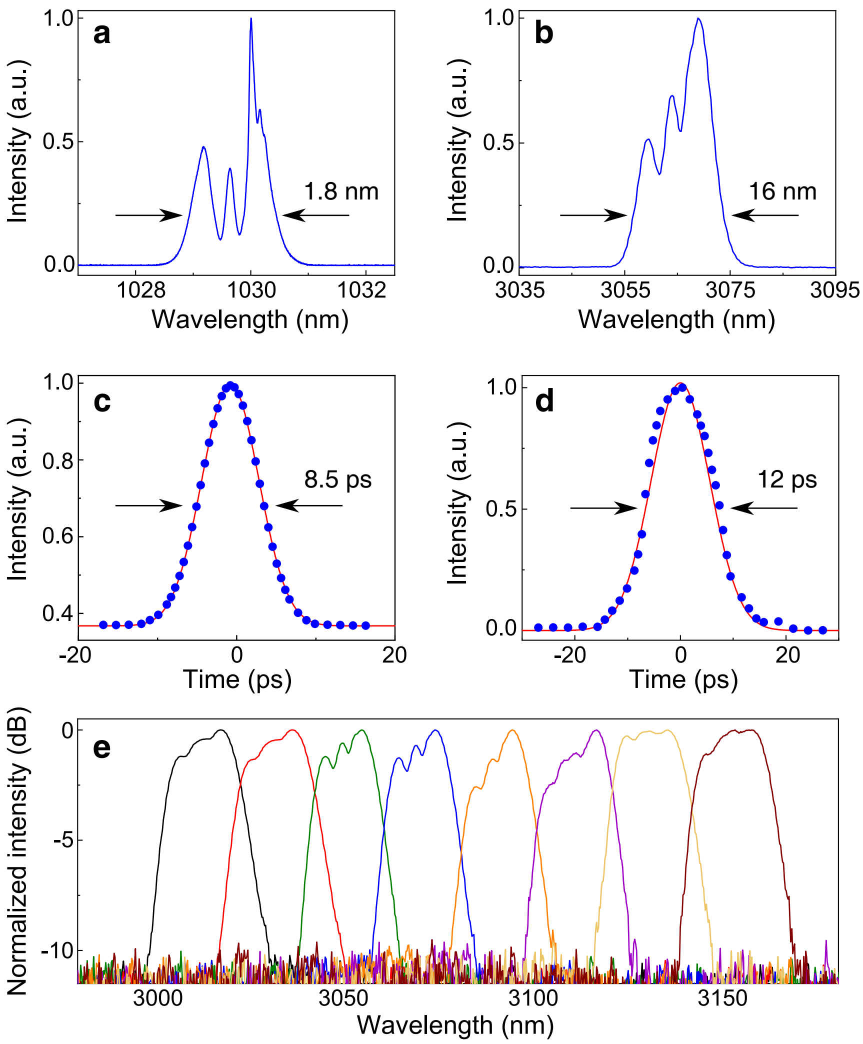

Supplementary Figure 2: Spectro-temporal characterization for the involved optical pulses. (a) Optical spectrum for the pump source. (b) Optical spectrum for the MIR signal. (c) Intensity profile of the pump pulse measured by the auto-correlator. (d) Cross-correlation trace between the signal and pump pulses. (e) Tunable MIR spectra while varying the output wavelength of the ECDL.

about two orders of magnitude lower than that by using a PPLN crystal for a given pump intensity. Thanks to high peak power in the pulsed excitation, the achieved efficiency is much higher than that in the CW-pumping scheme [5]. However, the conversion efficiency is relatively lower than that in our previous work [3]. One reason is the reduced pump intensity due to the expanded beam size. Another factor lies in the much shorter interaction length for each injecting beam at a given incidence angle. In our experiment, the modest pump power is operated slightly below the damage threshold of the fiber collimators within the delay line as shown in Supplementary Figure 1. This limitation can be eliminated by resorting to the free-space propagation for the pump beam. In this case, more than 6-W pump power can be utilized, which would increase the conversion efficiency up to 1%. Moreover, recent study indicates that a pump intensity up to 1 GW/cm^2 would lead to a high conversion efficiency of 20% [6].

The dark noise is another important figure of merit for the infrared detection. In contrast to the conventional MIR imager based on HgCdTe and InSb, the silicon-based cameras are featured with an extremely low dark current due to the much larger bandgap. The dark current of the EMCCD is specified to be about 10^{-4} electrons/pixel/second. Therefore, the background noise of the upconversion imaging system is mainly contributed by the pump-induced fluorescence. To characterize the imaging sensitivity, a noise equivalent energy can be defined as

$$\mathcal{N} = \frac{N_p \times M}{\eta_{\mathrm{QE}} \times G} \times \frac{1}{\eta_{\mathrm{conv}} \times \eta_{\mathrm{filter}}} \,, \tag{1}$$

where $N_p = 2.87\times10^{-7}$ count/pixel/pulse is the pump-induced noise current, $\eta_{\mathrm{QE}} = 80\%$ is the quantum efficiency at 771 nm for the EMCCD camera, $M = 4.93$ electron/count is the A/D conversion factor, $G = 1000$ is the gain factor, $\eta_{\mathrm{conv}} = 0.1\%$ is the conversion efficiency, and $\eta_{\mathrm{filter}} = 80\%$ is the total filtering efficiency. From the given values, $\mathcal{N} = 2.2\times10^{-6}$ photon/pixel/pulse can be calculated.

To evaluate the imaging performance, we have conducted the comparative investigation by using both the PPLN and CPLN crystals as shown in Supplementary Figures 3a and b, respectively. The resulting field of view (FOV) for the CPLN reaches to about 30°, which is almost ten times larger than that for the PPLN. In the experiment, the temperature of the PPLN crystal is stabilized at 50 °C to approach the phase-matching condition for a poling period of 20.9 μm. In contrast, the CPLN crystal is operated without the need of temperature stabilization, thus favoring simplicity

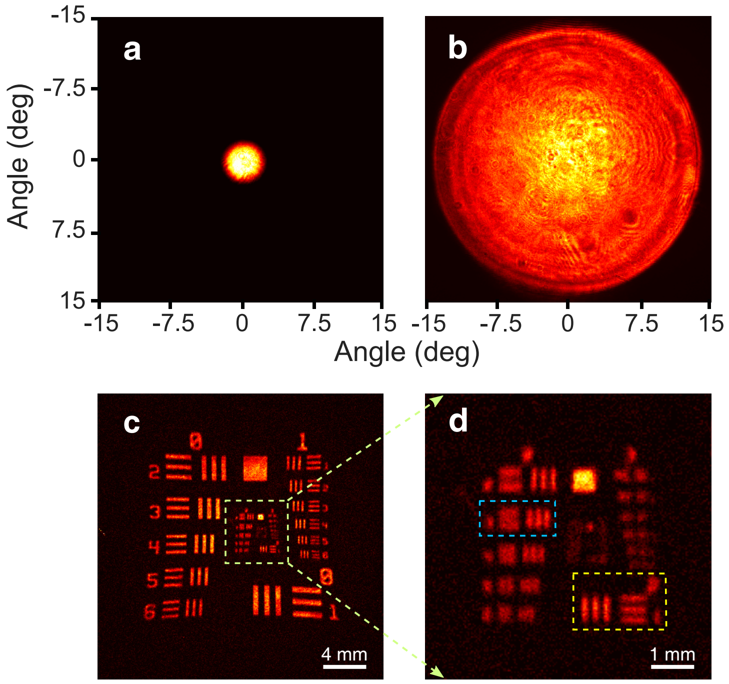

Supplementary Figure 3: Recorded field of view for the PPLN (a) and CPLN (b) crystals. Both crystals have a length of 10 mm. (c) Recorded image for a USAF-1951 resolution target. (d) Enlarged central part for the test chart. The elements in dashed boxes in blue and yellow indicate the resolution limits along the horizontal and vertical directions.

and robustness. Supplementary Figure 3c presents the recored image for a a USAF-1951 resolution target. The central part is then zoomed in to characterize the imaging resolution. As illustrated in Supplementary Figure 3d, the vertical resolution can be identified from the element within the yellow dashed box. The lines correspond to the first element of group 2 in the resolution chart, indicating a spatial resolution of 125 μm. The minimum resolvable element along the horizontal direction is denoted by the blue dashed box. The lines correspond to the third element of group 2, indicating a spatial resolution about 99 μm. The different resolutions in orthogonal directions are ascribed to the cropped pump beam within the nonlinear crystal.

During the upconversion imaging process, the components at different incidence angles are upconverted in different sections of the CPLN crystal. Particularly, the involved poling periods for the angles from 0 to 15 degrees correspond to the range between 19.5 and 21 μm, in part of the full range from 19 to 24 μm. In the experiment, two injection directions into the CPLN crystal have been tested for comparison, which exhibit similar imaging resolution and show no associated image aberration. One possible reason may lie in the homogeneous pump field along the crystal due to the large beam size. Further investigation could be conducted by numerally solving the three-dimensional coupled-wave equations.

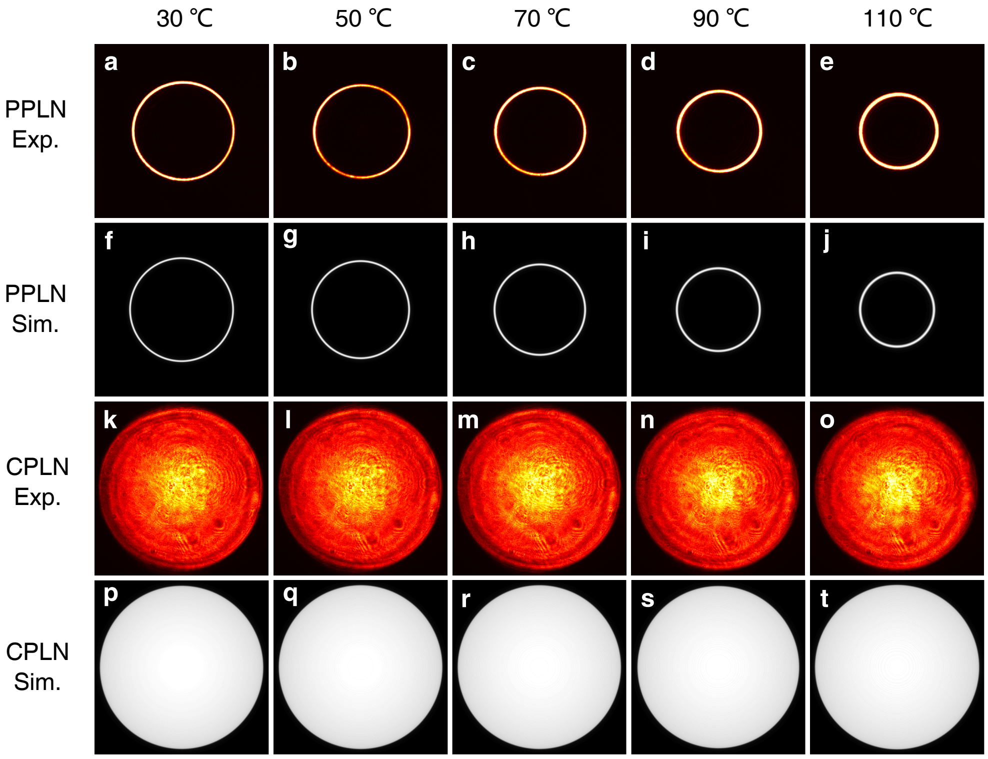

Supplementary Figure 4: Experimental observation (a-e) and theoretical simulation (f-j) for the conventional upconversion imaging with a PPLN. Experimental (k-o) and theoretical (p-t) results for the wide-field imaging with a CPLN crystal. The size for all the square images is 26.5 mm at the object plane.

Supplementary Note 3: Numerical simulation for upconversion imaging

The theoretical investigation for the upconversion imaging performance is based on the equations for the energy and momentum conversion laws in the quasi-phase-matching (QPM) configuration [7]. Particularly, the Sellmeier formula is adopted to valuate the refractive indexes for the three involved optical fields, which allows to calculate the phase mismatching at various parameter settings. At the presence of a given poling period and a crystal temperature, the angles for the MIR and SFG beams can be resolved with the constraint condition of the perfect phase matching. A program written in MATLAB is used to numerically optimize the related angles.

Supplementary Figure 4 represents the simulation results for the upconversion imaging based on the PPLN and CPLN crystals, which agree well with the experimental observation. It can be seen that the FOV in the conventional scheme with a PPLN crystal is severely limited by the phase-matching restriction. Concentric rings with a changed radius can be observed as varying the crystal temperature. In contrast, the use of the CPLN allows us to obtain a large FOV in one shot, which eliminates the need of parameter variation or data post-processing. Moreover, the exhibited immunity to the temperature change favors the simple and robust wide-field operation, which is desirable for subsequent applications. We note that the achieved FOV is technically limited by the entrance pupil of the 4f imaging system. Therefore, the theoretically allowed FOV can only be accessed by eliminating the limiting factors such as the aperture stop and crystal section in the upconversion imaging system.

Supplementary Note 4: High-speed MIR imaging at various frame rates

The frame rate is an important figure of merit for an imaging system in practical applications. A high refresh rate is usually required to capture transient events. However, the frame rate of the commercial MIR imagers is typically limited to below kHz due to the intrinsic response time of the material and the read-out speed of the electronics. In contrast, the current advance for silicon cameras has permitted the ultrafast acquisition at the MHz level. In this context, the upconversion technique has been introduced to extend the operation wavelength of the silicon sensor into the MIR regime. In previous demonstrations for the wide-field upconversion imaging, the frame rate was limited by the relatively long integration time, as required to accommodate the scanning cycle of the parameter ramping [8]. Such a restriction is eliminated in our implemented configuration, since the large FOV is realized in one shot without the need of the any parameter variation or data post-processing. Moreover, the superior imaging sensitivity benefits to obtain high-contrast images within a short exposure time at a modest illumination power.

In the experiment, we use a high-performance CMOS camera to implement an ultrafast MIR upconversion imaging, where a high-speed beam chopper is shot at ultra-high frame rates. The rotation frequency of the chopper is set to be 100 Hz, corresponding a line speed of 31.4 m/s for the slot at the edge. Supplementary Figures 5a,c,e,g present the recorded images at 15 k, 50 k, 100 k, and 216 k frames per second (fps), which correspond to the frame size of 640×640, 384×256, 256×160, and 128×80 pixels, respectively. Fewer pixels favor a shorter time for the frame readout

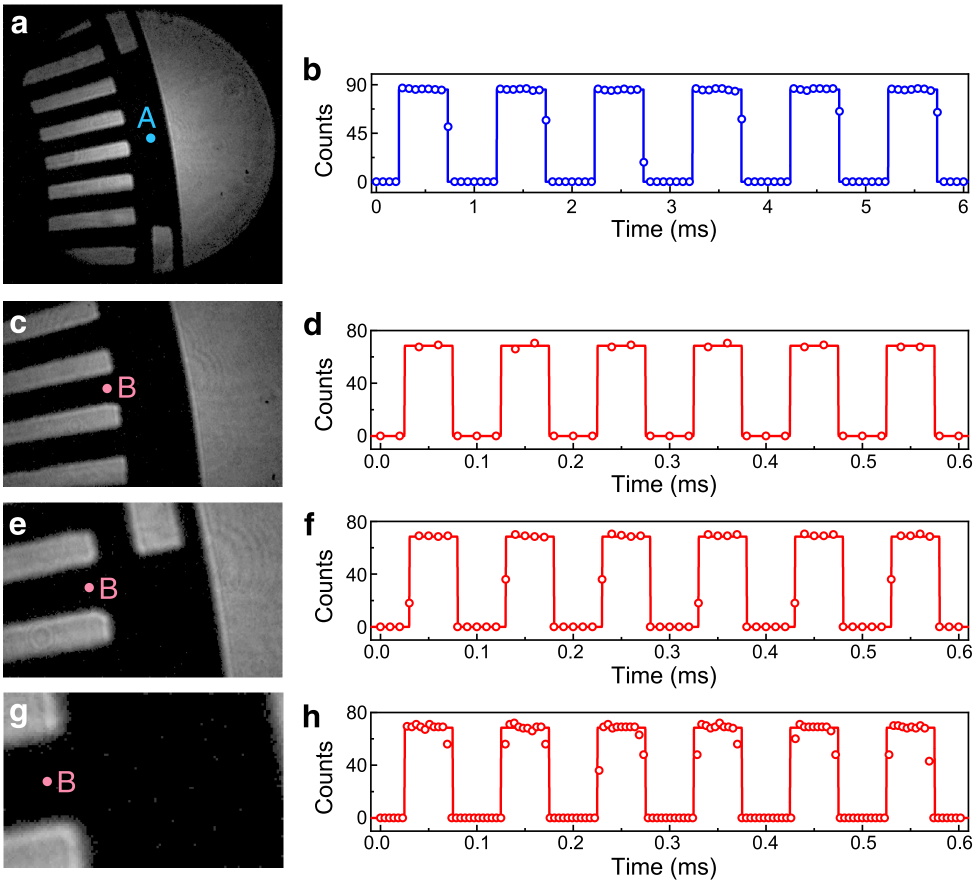

Supplementary Figure 5: Hight frame-rate MIR upconversion imaging. (a,c,e,g) illustrate the videography for a rapidly rotating chopper at frame rates of 15 k, 50 k, 100 k, 216 k fps. (b,d,f,h) present the intensity evolution for the pixels denoted with the dots. Note that the slots associated with the pixels A and B are specified to provide a chopping frequency of 1 and 10 kHz, respectively.

and the data manipulation in the camera. Hence, the reduction of pixel number is a common compromise to support the high-speed operation. The temporal evolutions for the intensity at the pixels A and B are given in Supplementary Figures 5b,d,f,h. The measured periods are consistent to the related chopping frequencies. As expected, denser points can be obtained at a higher frame rate, which can thus reveal more precise information for the transient process by a smaller-interval time stamp. Note that all the involve videos are recorded at the exposure time of 1.05 μs, which technically support a frame rate up to MHz.

Supplementary Note 5: Hyperspectral MIR imaging

The presented upconversion imaging system can immediately facilitate the hyperspectral MIR imaging by using a tunable source. In our experiment, the MIR illumination can be tuned from 3015 to 3170 nm, which is determined by the available tuning range for the ECDL. The corresponding upconversion images are illustrated in Supplementary Figure 6. These images are acquired at the

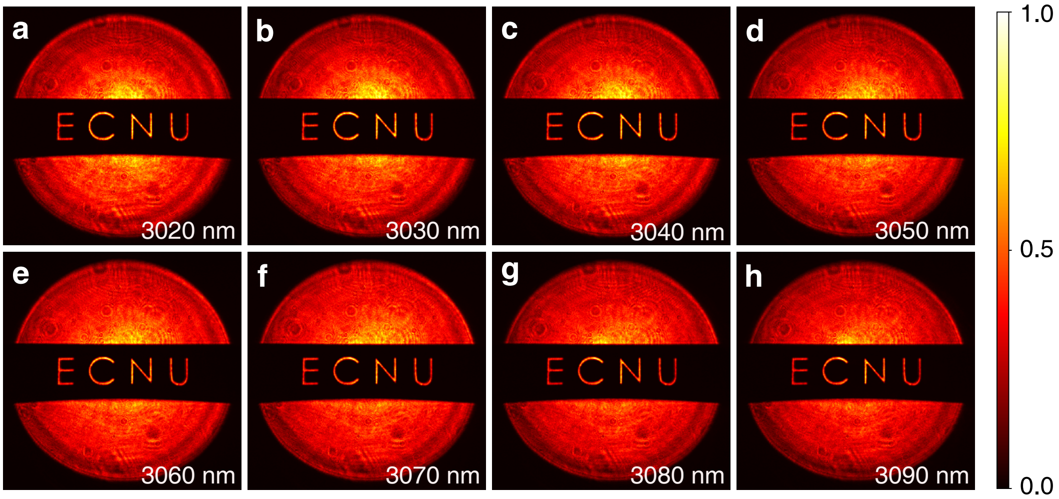

Supplementary Figure 6: Hyperspectral MIR imaging under the illumination at various wavelengths.

same parameter settings. Particularly, the CPLN is operated in the open air without the need of temperature stabilization. In principle, the CPLN crystal with a chirped range from 19 to 24 μm permits a phase-matching window from 2.7 to 5 μm. In this context, several tunable lasers covering different bands can be combined to extend the tuning range with the help of an optical switch module. Alternatively, the broadband MIR sources can be approached with the optical parametric oscillator or the quantum cascaded lasers.

The presented hyperspectral upconversion imaging is featured with a wide-field operation and ultrafast frame rate. Here the integration time for recording a monochromatic image is reduced to be about 1 μs, which is much shorter than the ms level in the previous work [8]. Consequently, the frame rate up to MHz can be obtained, thus substantially improving the speed of the volumetric data acquisition for the hyperspectral imaging.

Moreover, the rapid 3D MIR imaging with the chemically selectivity can be realized in combination with the time-resolving capability of the coincidence-pumping image upconverter. The imaging sensitivity here is significantly higher than the recent demonstration based on the two-photon absorption [9]. The proposed time-resolving spectral imaging would find promising applications in the low-light-level regime, such as tomographic imaging for opaque materials and biomedical examination without phototoxicity.

[1] Huang, K., Wang, Y., Fang, J., Chen, H., Xu, M., Hao, Q., Yan, M. & Zeng, H. Highly efficient difference-frequency generation for mid-infrared pulses by passively synchronous seeding. *High Power Laser Sci. Eng.* **9**, e4 (2021).

[2] Dam, J. S., Tidemand-Lichtenberg, P. & Pedersen, C. Room-temperature mid-infrared single-photon spectral imaging. *Nat. Photon.* **6**, 788-793 (2012).

[3] Wang, Y., Fang, J., Zheng, T., Liang, Y., Hao, Q., Wu, E., Yan, M., Huang, K. & Zeng, H. Mid-infrared single-photon edge enhanced imaging based on nonlinear vortex filtering. *Laser Photon. Rev.*, 2100189

(2021).

[4] Huang, K., Wang, Y., Fang, J., Kang, W., Sun, Y., Liang, Y., Hao, Q., Yan, M. & Zeng, H. Mid-infrared photon counting and resolving via efficient frequency upconversion. *Photon. Res.* **9**, 259-265 (2021).

[5] Friis, S. & Høgstedt, L. Upconversion-based mid-infrared spectrometer using intra-cavity LiNbO 3crystals with chirped poling structure. *Opt. Lett.* **44**, 4231(2019).

[6] Mrejen, M., Erlich, Y., Levanon, A. & Suchowski, H. Multicolor time-resolved upconversion imaging by adiabatic sum frequency conversion. *Laser Photon. Rev.* **14**, 2000040 (2020).

[7] Tidemand-Lichtenberg P., Dam J. S., Andersen H. V., Høgstedt L. & Pedersen C. Mid-infrared upconversion spectroscopy. *J. Opt. Soc. A. B* **33**, D28 (2016).

[8] Junaid, S., Chaitanya Kumar, S., Mathez, M., Hermes, M., Stone, N., Shepherd, N., Ebrahim-Zadeh, M., Tidemand-Lichtenberg, P. & Pedersen, C. Video-rate, mid-infrared hyperspectral upconversion imaging. *Optica* **6**, 702-708 (2019).

[9] Potma, E. O., Knez, D., Chen, Y., Davydova, Y., Durkin, A., Fast, A., Balu, M., Norton-Baker, B., Martin, R. W., Baldacchini, T. & Fishman, D. A. Rapid chemically selective 3D imaging in the mid-infrared. *Optica* **8**, 995 (2021).
