# Supplementary material for: Wide-field mid-infrared single-photon upconversion imaging
Source: Nat Commun. 2022 Feb 28;13:1077. doi: 10.1038/s41467-022-28716-8 (PMC8885736; doi:10.1038/s41467-022-28716-8)
Supplement: Supplementary file 3 — Description of Additional Supplementary Files [file 41467_2022_28716_MOESM3_ESM.docx]

**Description of Additional Supplementary Files**

**File Name:** Supplementary Video 1

**Description:** Real-time transmission imaging for a campus ID card under the MIR illumination. The translational speed of the card at the horizontal direction is about 2.5 mm/s. Movie is acquired with the 20-ms exposure time at the frame rate of 20 fps.

**File Name:** Supplementary Video 2

**Description:** MIR videography of a high-speed beam chopper at ultra-high frame rates. The rotation frequency of the chopper is set to be 100 Hz, corresponding a line speed of 31.4 m/s for the slot at the edge. Movies are acquired with the 1.05-μs exposure time at 15, 50, 100, and 216 kfps.

**File Name:** Supplementary Video 3

**Description:** Recorded video for the MIR three-dimensional imaging by scanning the time delay between the signal and pump pulses. The speed of the translational stage in the delay line is set to 1 mm/s. Movie is acquired with the 100-ms exposure time at 7 fps.
